# Reconstruction of a human cornea by the self-assembly approach of tissue engineering using the three native cell types

**Published:** 2010-10-29

**Authors:** Stéphanie Proulx, Jeanne d’Arc Uwamaliya, Patrick Carrier, Alexandre Deschambeault, Caroline Audet, Claude J. Giasson, Sylvain L. Guérin, François A. Auger, Lucie Germain

**Affiliations:** 1 Centre LOEX de l'Université Laval, Génie tissulaire et régénération: LOEX - Centre de recherche FRSQ du Centre hospitalier *affilié* universitaire de Québec, Québec, QC, Canada; 2 Département d’Oto-rhino-laryngologie et d’Ophtalmologie, Université Laval, Québec, QC, Canada; 3 Département de Chirurgie, Faculté de médecine, Université Laval, Québec, QC, Canada; 4 École d’Optométrie, Université de Montréal, Montréal, QC, Canada

## Abstract

**Purpose:**

The purpose of this study was to produce and characterize human tissue-engineered corneas reconstructed using all three corneal cell types (epithelial, stromal, and endothelial cells) by the self-assembly approach.

**Methods:**

Fibroblasts cultured in medium containing serum and ascorbic acid secreted their own extracellular matrix and formed sheets that were superposed to reconstruct a stromal tissue. Endothelial and epithelial cells were seeded on each side of the reconstructed stroma. After culturing at the air-liquid interface, the engineered corneas were fixed for histology and transmission electron microscopy (TEM). Immunofluorescence labeling of epithelial keratins, basement membrane components, Na^+^/K^+^-ATPase α1, and collagen type I was also performed.

**Results:**

Epithelial and endothelial cells adhered to the reconstructed stroma. After 10 days at the air-liquid interface, the corneal epithelial cells stratified (4 to 5 cell layers) and differentiated into well defined basal and wing cells that also expressed Na^+^/K^+^-ATPase α1 protein, keratin 3/12, and basic keratins. Basal epithelial cells from the reconstructed epithelium formed many hemidesmosomes and secreted a well defined basement membrane rich in laminin V and collagen VII. Endothelial cells formed a monolayer of tightly-packed cells and also expressed the function related protein Na^+^/K^+^-ATPase α1.

**Conclusions:**

This study demonstrates the feasibility of producing a complete tissue-engineered human cornea, similar to native corneas, using untransformed fibroblasts, epithelial and endothelial cells, without the need for exogenous biomaterial.

## Introduction

The cornea constitutes the interface between the eye and the surrounding environment and provides 75% of the eye’s refractive power. It consists of three layers: the outermost epithelium, which is in direct contact with the environment, the stroma, and the innermost endothelium cell layer. The corneal epithelium is pluristratified, made out of cuboidal basal cells that gradually become flatter as they differentiate toward the surface [1,2]. Basal cells rest on a basement membrane to which they attach by the formation of hemidesmosomes. The corneal epithelial basement membrane is composed of collagen types IV, VII, XII, and laminin [3]. This basement membrane lies on the Bowman’s membrane, a less organized form of corneal stroma. The corneal stroma represents 90% of the corneal thickness [1,2] and consists of regularly-arranged collagen fibers (mainly type I collagen) along with sparsely distributed interconnected keratocytes. These cells produce and maintain the stromal matrix. The corneal endothelium is a monolayer of flattened cells that face the anterior chamber of the eye [1,2]. The main function of these cells is to pump fluid out of the corneal stroma, allowing the cornea to remain optically clear. Numerous studies have demonstrated that the active transport systems of the corneal endothelium require sodium and bicarbonate ions and is driven by the sodium pump, Na^+^/K^+^-adenosine triphosphatase (Na^+^/K^+^-ATPase) [4-7].

In vitro studies aimed at reconstructing the corneal epithelium have shown that culturing cells at the air-liquid interface (air-lifting) resulted in stratification and differentiation of corneal epithelial cells. The air-lifted epithelium was positive for keratin 3 (K3) [8-13], a well known marker of corneal epithelial differentiation [14]. This stratified reconstructed epithelium was also shown to have improved transepithelial electrical resistance and active ion transport properties similar to those of native corneas [10]. Epithelial cells need to be cultured either on a support or on a stromal substitute to be air-lifted. So far, there have been reports of air-lifting epithelial cells using uncoated inserts [15], inserts coated with collagen [9], or coated with a mixture of collagen, fibronectin, and laminin [10], amniotic membranes [11,16], collagen gels [17-20], collagen sponges [21] or hydrogels [22,23].

A particularly interesting stromal support derivative developed in our laboratory consists in inducing stromal fibroblasts to secrete and lay down their own extracellular matrix in vitro, which then form sheets on which epithelial cells can be cultured and air-lifted. This concept, called the self-assembly approach, has been used in our laboratory to produce skin [24] and corneal substitutes constituted of the two outmost cell layers [12,25]. Our human corneal epithelium model has allowed us to study wound healing in a way that could not be achieved using native human corneas. Comparatively to other developed models, our procedure offers a completely biologic cellular environment, using living fibroblasts and epithelial cells, without adding any synthetic material.

The reconstruction of corneas in vitro using all three corneal cell types has been previously reported [21,26-37]. Most studies have either used immortalized cell lines [21,26,27,30,33,36] or animal cells [28,29,31,34,35], both of which are not representative models of normal human corneas. Since it has been shown that adding endothelial cells improved epithelial differentiation of reconstructed corneas [36,37] and because our laboratory has developed the expertise for isolating and cultivating endothelial cells [38-40], we improved our previous model by reconstructing a complete cornea, using all three corneal cell types, and compared this new human corneal model with native corneas.

## Methods

### Isolation and culture of human corneal cells

This study was conducted according to our institution’s guidelines and the Declaration of Helsinki. Normal human corneas, unsuitable for transplantation or following surgical resection, were obtained from our local Eye Bank (Banque d’Yeux Nationale, Québec, Québec, Canada) and the department of Ophthalmology at the Centre Hospitalier de l’Université de Montréal (CHUM; Dr Christine Corriveau). Isolation and culture of human limbal epithelial cells was performed using four different donor eyes (aged 3, 12, 51, and 84 years-old), as previously described [20]. Briefly, corneas were incubated in 2 mg/ml dispase II (Roche Diagnostics, Laval, Qc, Canada) in HEPES buffer (MD Biomedicals, Montreal, Québec, Canada) overnight at 4 °C. The epithelium of the limbal region was removed and then seeded in tissue culture flasks (BD Biosciences, Mississauga, Ontario, Canada) with irradiated murine Swiss-3T3 fibroblasts (ATCC, Rockville, MD) in the described limbal epithelial cells culture medium [20]. Human limbal epithelial cells were used between passages 3 and 5. Isolation and culture of human corneal endothelial cells was performed using three different donor eyes (aged 11 months, 43, and 50 years-old), as previously described in Zhu and Joyce [41]. Briefly, strips of Descemet’s membrane were peeled-off and incubated overnight at 37 °C in growth medium. After centrifugation, they were incubated one hour in 0.02% EDTA (Sigma, Oakville, Ontario, Canada) and the loosened cells were detached from Descemet’s membrane by passing several times through a flamed-polished pipet. Cells were then centrifuged and resuspended in fresh medium [41]. Corneal endothelial cells were used between passages 1 and 5. Human corneal fibroblasts were isolated from the stromal portion of a cornea (aged 26 days-old) left after dispase digestion and removal of both the endothelium and epithelium, and cultured as previously described [42,43]. Briefly, stromal explants were seeded in culture flasks and cultured in fibroblast culture medium consisting of DMEM supplemented with 10% fetal calf serum (Hyclone, Logan, UT), 25 µg/ml gentamycin sulphate (Schering, Pointe Claire, Québec, Canada) and 100 IU/ml penicillin G (Sigma). Human skin fibroblasts were obtained from the dermal portion of adult breast skin (of one 23 years-old donor) and were cultured as previously described [25,44,45]. Briefly, the skin was incubated overnight at 4 °C in a thermolysin (Sigma) solution (500 µg/ml in HEPES buffer, pH 7.4). The epidermis was peeled from the dermis and incubated for 30 min in a trypsin-EDTA solution (0.05% trypsin; MD Biomedicals, and 0.01% EDTA; JT Baker, Phillipsburg, NJ) to dissociate epithelial cells. The dermis was incubated for 3 h in a collagenase H (Roche Diagnostics) solution (0.125 IU/ml in the fibroblast culture medium) to recover fibroblasts.

### Reconstruction of human corneas using a self-assembled stromal substitute

Corneal and dermal fibroblasts were separately seeded at 80 cells/mm^2^ and cultured in fibroblast growth medium supplemented with 50 µg/ml ascorbic acid (Sigma) for 28–35 days. Ascorbic acid allows fibroblasts to secrete and lay down their own extracellular matrix, forming thick sheets which can be superposed to reconstruct a corneal stroma [12]. Corneal endothelial cells were then seeded on top of this self-assembled stroma and cultured for two to seven days in the endothelial growth medium, after which they were turned over a plastic ring. Limbal epithelial cells were seeded on the other side and cultured in epithelial cell medium, supplemented with 50 µg/ml ascorbic acid (Sigma), immerged for seven days, then lifted at the air-liquid interface for ten days. Using the different cell populations of epithelial and endothelial cells, five different corneas were reconstructed in duplicates. Tissue-engineered corneas were also reconstructed without endothelial cells.

### Histological analysis

Biopsies were fixed in 1% Histochoice (Amresco, Solon, OH) and embedded in paraffin. Cross-sections (5 µm) were stained (Masson’s trichrome staining) and analyzed by light microscopy. Thickness was measured using AxioVision 4.8.1 (Carl Zeiss, Toronto, Ontario, Canada).

### Indirect immunofluorescence analysis

Specimens were embedded in Optimal Cutting Temperature compound (OCT; Somagen, Edmonton, Alberta, Canada) frozen in liquid nitrogen and then stored at −70 °C until use. An indirect immunofluorescence assay was performed on acetone-fixed cryosections (5 µm) of engineered corneas as previously described [20]. Briefly, sections were incubated with antibodies, diluted in phosphate buffered saline (PBS: 137 mM NaCl, 2.7 mM KCl, 6.5 mM Na_2_HPO_4_, 0.9 mM CaCl_2_, 0.48 mM MgCl_2_ containing 1% BSA) at room temperature for 45 min (primary antibodies) or for 30 min (conjugated antibodies). Primary antibodies used were: anti-keratin-AE3 (MP Biomedicals, Solon, OH), anti-keratin 3/12 (AE5; MP Biomedicals), anti-human collagen 1 (Calbiochem, Montreal, Quebec, Canada), anti-human collagen VII (Millipore, Nepean, Ontario, Canada), anti-laminin V chain γ2 (Millipore), anti-laminin (Sigma), anti-Na^+^/K^+^-ATPase α1 (clone C464.6; Millipore) and anti-ZO-1 (ZMD-436; Invitrogen, Burlington, Ontario, Canada). Goat anti-mouse IgG H+L antibodies conjugated with Alexa 594 (Invitrogen) and chicken anti-rabbit antibodies conjugated with Alexa 594 (Invitrogen) were used as secondary antibodies. Cell nuclei were counterstained with Hoechst reagent 33258 (Sigma). Negligible background was observed for controls (primary antibodies omitted). Fluorescence was observed using an epifluorescence microscope (Eclipse TE-2000U inverted microscope; Nikon, Mississauga, Ontario, Canada) and slides photographed with a numeric charge-coupled device camera (ORCA-ER; resolution 1344×1024; Hamamatsu, Bridgewater, NJ).

### Electron microscopy analysis

Samples were fixed in 2.5% glutaraldehyde (Canemco, Lakefield, Québec, Canada) and processed for transmission electron microscopy (TEM), as described [38]. Briefly, the glutaraldehyde-fixed cornea was washed in cacodylate buffer, postfixed in 1% osmium tetraoxide, stained with 0.5% uranyl acetate, dehydrated in a graded series of ethanol solutions, and embedded in Poly/Bed 812. Thin sections were processed and visualized using a JEOL JEM-1230 (Tokyo, Japan) transmission electron microscope at 80 kV.

## Results

### Morphological characterization of tissue-engineered corneas

**Figure 1:**
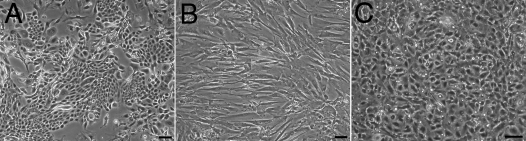
Morphology of human corneal cells cultured as monolayers on a plastic substrate. **A**: Corneal epithelial cells co-cultured with an irradiated 3T3 feeder layer. B: Stromal fibroblasts. **C**: Corneal endothelial cells. Bar, 100 µm.

The three corneal cell types were first isolated and cultured separately on plastic. Figure 1 shows the typical cell morphology of all three cell types. Corneal epithelial cells formed large colonies of small cells (Figure 1A), stromal cells showed a fibroblastic morphology (Figure 1B), and endothelial cells had a polygonal shape (Figure 1C).

**Figure 2:**
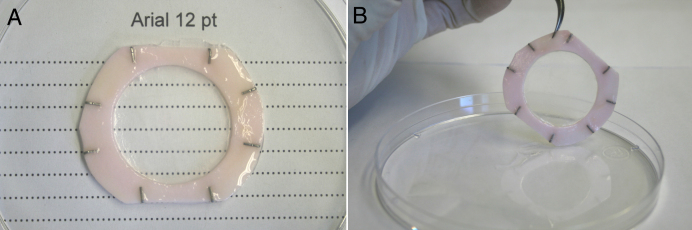
Macroscopic aspect of the bioengineered cornea. **A**: Dots written in arial 12 pt font can easily be seen through the bioengineered cornea. **B**: The bioengineered cornea possesses a slight haze.

**Figure 3:**
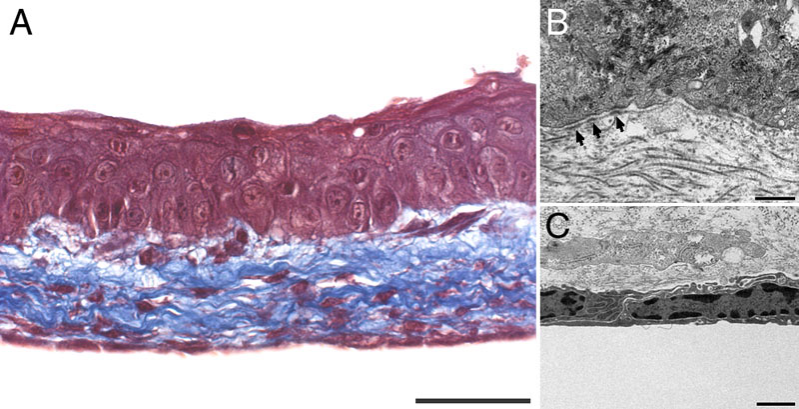
Tissue-engineered human cornea. **A**: Histology (Masson’s Trichrome staining) of the tissue-engineered cornea, showing a well differentiated epithelium on top, and a monolayer of endothelial cells underneath, both adhered to the self-assembled stromal matrix. **B**: Transmission electron microscopy of the epithelial basal membrane showing many hemidesmosomes (arrows). **C**: Transmission electron microscopy of the corneal endothelium, showing a monolayer of flattened cells. The bar in **A** equals 50 µm, in **B** equals 1 µm, and in **C** equals 0.5 µm.

Corneal endothelial and epithelial cells were then seeded and cultured on each side of the self-assembled stroma, composed of natural matrix secreted by the human fibroblasts. The macroscopic aspect reveals that the complete bioengineered cornea is clear enough to be able to read through it, having only a slight haze (Figure 2). Figure 3A shows the histological staining of the tissue-engineered corneas. The thickness of the stromal substitute was 35.0±9.5 µm. Interestingly, when endothelial cells were not added, the stromal substitute had a thickness of 55.0±11.9 µm. After ten days at the air-liquid interface, the corneal epithelium was composed of four to five cell layers. The well stratified corneal epithelium possessed cuboidal basal cells, which flattened as they differentiated into superficial cells. There was no evidence of typical staining for Descemet’s or Bowman’s membrane. In the histology cross sections, the epithelia from corneas reconstructed either with or without endothelial cells had similar number of cell layers and reached an identical level of differentiation (not shown). Using TEM, the basement membrane along the epithelium-stromal junction was easily visible, possessing many hemidesmosomes that attach basal cells to the self-assembled stromal matrix (Figure 3B). Endothelial cells adhered and formed a continuous monolayer covering the entire surface of the reconstructed stroma (Figure 3A,C). TEM confirmed that the endothelial cells formed a tight monolayer of flattened cells (Figure 3C).

### Collagenous matrix of the stromal substitute reconstructed using the self-assembly approach

**Figure 4:**
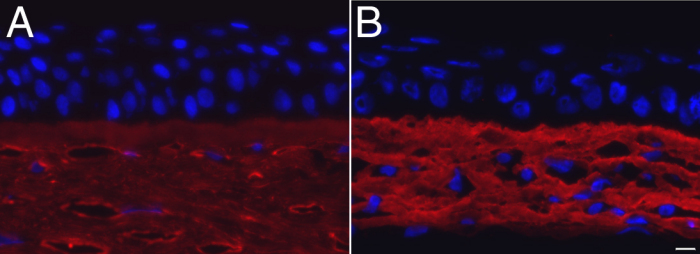
Collagen type I immunofluorescence staining. Strong collagen type I staining (in red) was found in both the native (**A**) and reconstructed stromas (**B**). Nuclei were counterstained with Hoechst (in blue). Bar, 10 µm.

Immunofluorescent detection of collagen type I was used to assess the presence of this protein in the reconstructed stroma. A strong expression of collagen type I was observed in both the native (Figure 4A) and the reconstructed corneal stroma (Figure 4B).

### Epithelial keratins and basement membrane components

**Figure 5:**
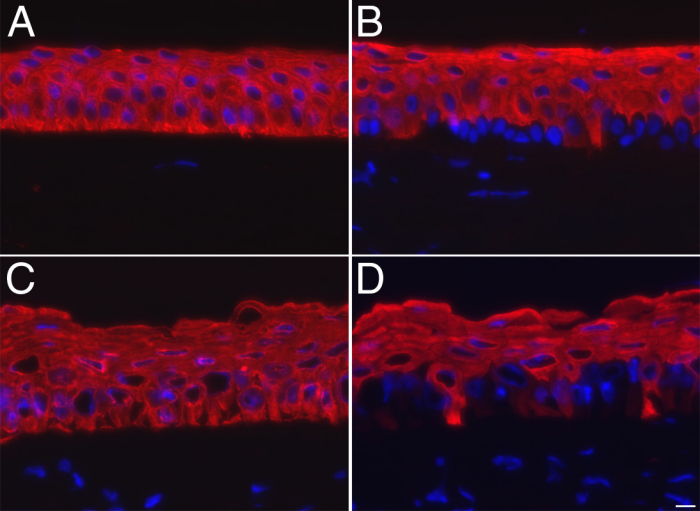
Epithelial keratins. **A**, **B**: Native human corneas. **C**, **D**: Tissue-engineered corneas. **A**, **C**: Immunofluorescence staining of basic keratins (in red). **B**, **D**: Immunofluorescence staining of keratin 3/12 (in red). Nuclei were counterstained with Hoechst (in blue). Bar, 10 µm.

The pan-cytokeratin AE3 antibody detects all known basic keratins whereas AE5 is an antibody specific for keratin 3/12, a well known marker of corneal epithelial cells [14]. Basic keratins were detected throughout all epithelial cell layers from both native human (Figure 5A) and tissue-engineered corneas (Figure 5C). K3/12 was detected in some basal cells but mostly in suprabasal cells from the tissue-engineered corneas (Figure 5D). A similar K3/12 staining pattern was observed in native human corneas (Figure 5B).

**Figure 6:**
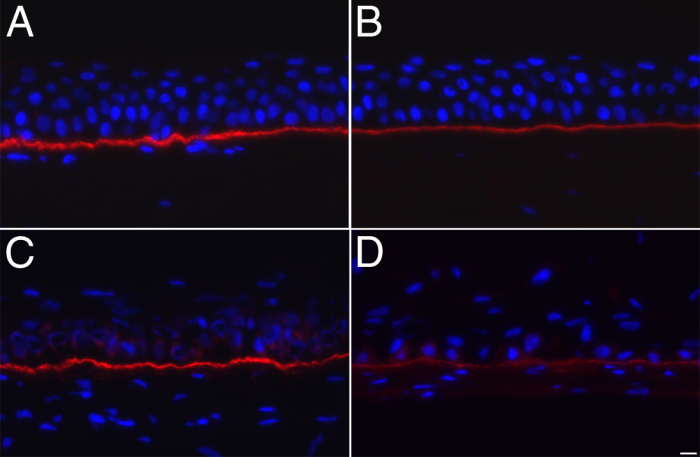
Epithelial basement membrane components. **A**, **B**: Native human corneas. **C**, **D**: Tissue-engineered corneas. **A**, **C**: Immunofluorescence staining of collagen VII (in red). **B**, **D**: Immunofluorescence staining of laminin V (in red). Nuclei were counterstained with Hoechst (in blue). Bar, 10 µm.

The secretion of laminin V and collagen type VII by corneal epithelial cells cultured on the self-assembled stroma was next evaluated by immunofluorescence (Figure 6). Both basement membrane components were present as a continuous line along the epithelium-stromal junction (Figure 6C,D). However, the staining intensity of collagen type VII in the tissue-engineered corneas (Figure 6D) was weaker than that observed in the basement membrane of the corneal epithelium from native corneas (Figure 6B). Comparison of the expression of keratin and basement membrane components revealed no significant difference between tissue-engineered corneas reconstructed with all three cell types and those without endothelial cells (data not shown).

### Na^+^/K^+^-ATPase expression in the epithelial and endothelial monolayer of tissue-engineered corneas

**Figure 7:**
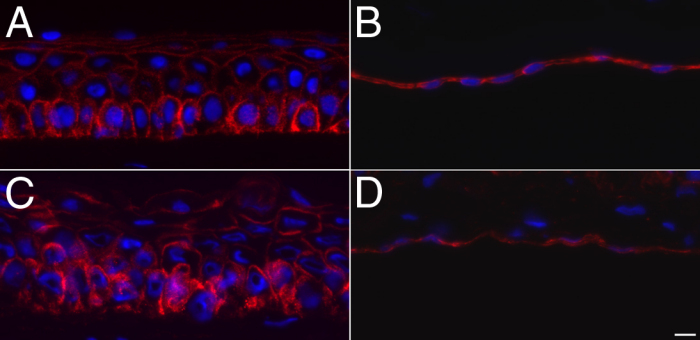
Immunofluorescence staining of Na^+^/K^+^-ATPase α1. **A**, **C**: The corneal epithelium has a bottom-up decreasing gradient of expression of the Na^+^/K^+^-ATPase pumps (in red) from the basal layer toward the wing cells in both native corneas (**A**) and reconstructed corneas (**C**). **B**, **D**: Immunofluorescence staining of Na^+^/K^+^-ATPase α1 of the corneal endothelium in both native (**B**) and reconstructed corneas (**D**). Nuclei were counterstained with Hoechst (in blue). Bar, 10 µm.

As seen in Figure 7, Na^+^/K^+^-ATPase expression was detected in both the native and the reconstructed corneal epithelium, with basal cells having a stronger staining than suprabasal cells. Detection of Na^+^/K^+^-ATPase α1 protein, heavily expressed by native endothelial cells, was also used to examine the reconstructed endothelium cultured on the tissue-engineered corneas. The staining revealed a continuous monolayer of endothelial cells, having a staining pattern for Na^+^/K^+^-ATPase α1 similar to the native corneal endothelium (Figure 7D,B, respectively).

## Discussion

Tissue engineering is an attractive approach for the production of tissues in vitro. Our results demonstrate the feasibility of reconstructing a full thickness human cornea using native corneal cells. We show that these tissue-engineered corneas have a structure similar to native human corneas.

Reconstructing a functional tissue in vitro first requires cells of high quality. The presence of stem cells with their self-renewing ability favors the production and regeneration of the epithelium. Cell isolation and culture are the initial steps for the production of tissue-engineered substitutes. Furthermore, cells have to be grown under appropriate conditions so that the tissue reconstructed with the cultured cells maintains functional and regenerative capacities. Previous studies have demonstrated that epithelial stem cells co-cultured with irradiated fibroblasts preserve their stem cell characteristics [46,47]. They produce large colonies constituted of small cells that possess a high nucleus to cytoplasm ratio. These characteristics are typical of less differentiated cells. It is also known that corneal endothelial cells need optimal medium conditions to preserve their initial morphology [48]. Since the culture medium of human corneal endothelial cells has been optimized [41], it is now possible to culture these cells for many passages while maintaining their characteristic polygonal morphology, as seen in Figure 1.

We cultivated corneal and dermal fibroblasts in the presence of serum and ascorbic acid, promoting extracellular matrix assembly and allowing the formation of thick sheets of collagenous tissue, as previously demonstrated by our [12,43] as well as other teams [49,50]. The epithelialized stromal substitute is both transparent and has ultraviolet-absorbance characteristics similar to those of native human corneas [25]. In the present study, we have shown that this biocompatible, self-assembled living stromal substitute allowed for the adhesion and growth of corneal epithelial and endothelial cells. This 3D-three-layer human corneal substitute could be further improved by growing epithelial and endothelial cells on an aligned stromal matrix. We recently reported that cultivating corneal fibroblasts on a patterned substrate allows for the production of an aligned stromal matrix, which was shown to be more transparent than disorganized ones [51], further enhancing the quality of the reconstructed stromal substitute.

Because of its importance in vision and in protecting the eye against external injuries, the corneal epithelium needs to be well stratified. It must also express keratins that ensure its stability and integrity, while allowing the anchorage between the epithelium and the basement membrane and between epithelial cells themselves. As others who reported that stratification and differentiation was prompted by lifting epithelial cells at the air-liquid interface [8-13], we also showed that our epithelium stratified and differentiated as it naturally occurs within native corneas. K3/12 staining was mostly found in suprabasal cells. The fact that basal cells are K3/12 negative suggests the presence of stem cells in the epithelial basal layer of our reconstructed corneas. As previously demonstrated, this keratin is not expressed by undifferentiated cells (limbal stem cells) and is mainly located in the suprabasal layers in native limbus [12,14,52]. This is a strong point to indicate that the engineered epithelium could maintain a continuous renewal capacity which is ensured either by undifferentiated or less differentiated basal cells. The tissue-engineered corneas also possessed a well defined and continuous basement membrane. Our results agree with those of other published studies that demonstrated the formation of an epithelial basement membrane in reconstructed corneas in vitro [36,53]. Although previous studies reported that the presence of endothelial cells improved the structure of the basal membrane and enhanced the differentiation of the epithelium [31,36,37], our results could not show any significant differences in the epithelium’s differentiation properties whether endothelial cells where present or not in the reconstructed corneas. Since these studies used exogenous collagen [31,36,37] and immortalized endothelial cells [36], we hypothesize that our model, using native cells which secreted a completely natural stromal matrix, provided a closer physiologic condition for the epithelial cells that also promoted a better differentiation than achieved using collagen sponges or gel matrices and/or transformed cells.

The gradient staining of the Na^+^/K^+^-ATPase α1 protein that is typical of the epithelium from native human corneas (the basal cells have a strong staining that progressively decreases from basal cells to the upper, more differentiated cell layers) could also be observed in the epithelia of our tissue-engineered corneas. This gradient staining has also recently been reported in native and reconstructed skin [54]. Our results also demonstrate that the endothelial cell monolayer expressed the important function-related protein Na^+^/K^+^-ATPase α1, a result in agreement with other previously reported studies whose reconstructed endothelium also expresses Na^+^/K^+^-ATPase [38,39,55-61]. Further experiments will be necessary to assess the functionality of the pumps.

This study supports the notion that a full thickness human tissue-engineered cornea can be produced in vitro using native corneal cells. This fully biologic model, developed from untransformed human corneal cells, shows histological properties close to those of a human native cornea with some minor deficiencies that could be improved upon. Although the tissue-engineered stroma was thinner than native stroma in the present study, its thickness could be increased by superposing a greater number of stromal sheets using a similar methodology. Thicker tissue-engineered substitutes were produced for skin and cardiac valves [62,63]. This novel corneal model can well be used for studies of multiple corneal pathologies such as abrasions, wound healing, endothelial and stromal dystrophies, or for pharmacological and toxicological studies. Tissue-engineered corneas reconstructed using the self-assembly approach will surely benefits from the ongoing progresses in tissue reconstruction and hopefully should soon be available clinically for the treatment of various corneal disorders.
